# Forgotten but Not Gone: Pediatric Scrub Typhus in an Urbanizing Tropical Region

**DOI:** 10.7759/cureus.114733

**Published:** 2026-08-18

**Authors:** Dac Cuong Nguyen, Hai Trieu Giang, Dinh Mai Vu, Viet Van Nguyen

**Affiliations:** 1 Pediatrics, Rostov State Medical University, Rostov-on-Don, RUS; 2 Pediatrics, Hai Phong University of Medicine and Pharmacy, Haiphong, VNM; 3 Neonatology, Haiphong Children's Hospital, Haiphong, VNM

**Keywords:** orientia tsutsugamushi, pediatric infectious diseases, prolonged fever, scrub typhus, tropical medicine

## Abstract

Scrub typhus is an acute febrile illness caused by *Orientia tsutsugamushi*, transmitted to humans through the bite of infected larval trombiculid mites. Its nonspecific clinical presentation often leads to delayed diagnosis and treatment, increasing the risk of severe complications. As urbanization progresses and awareness declines, clinicians may overlook this diagnosis, particularly when characteristic findings such as an eschar are absent or have been resolved. We report the case of an 11-year-old boy from Vietnam who was admitted with recurrent fever persisting for two weeks, occasionally accompanied by abdominal distension. Further history revealed a previous insect bite that had resolved before admission and recent cases of scrub typhus among individuals living in the same neighborhood. Given the epidemiological context, targeted investigations were performed, leading to the identification of *Orientia tsutsugamushi *infection and confirmation of scrub typhus. Appropriate antibiotics resulted in significant clinical improvement. Thorough assessment of epidemiological exposure and early diagnostic testing are essential for timely diagnosis and treatment, thereby reducing morbidity and preventing potentially life-threatening complications.

## Introduction

Scrub typhus is an acute febrile illness caused by *Orientia tsutsugamushi *(formerly *Rickettsia tsutsugamushi*), an obligate intracellular Gram-negative bacterium transmitted to humans through the bite of infected larval *Leptotrombidium *mites [1]. The disease is endemic throughout the so-called "Tsutsugamushi Triangle", a region extending across the Asia-Pacific area, including Southeast Asia, East Asia, South Asia, northern Australia, and parts of the western Pacific islands. It is estimated that approximately one million cases occur annually, while nearly one billion people worldwide are at risk of infection [2,3].

If left untreated, scrub typhus may result in substantial morbidity and mortality, with reported case fatality rates ranging to as high as 30%, depending on geographic location, healthcare accessibility, and disease severity [4]. The clinical manifestations vary widely, ranging from a self-limited febrile illness to severe multisystem involvement. Neurological complications have been increasingly recognized and include meningitis, encephalitis, ischemic stroke, seizures, Guillain-Barré syndrome, and myositis. Although these complications are more commonly reported in adults, pediatric patients may also experience severe disease when diagnosis and treatment are delayed.

In endemic regions, scrub typhus is an important cause of acute undifferentiated fever in children. Several studies conducted throughout Asia have demonstrated that pediatric patients account for a substantial proportion of cases, particularly in rural, suburban, and mountainous areas. In Vietnam, scrub typhus has been reported to account for approximately 3.5% of hospital admissions for acute undifferentiated febrile illness (AUFI) [5], highlighting its significance as a public health concern and the need for increased clinical awareness among healthcare providers.

Given its nonspecific clinical presentation and the frequent absence of characteristic findings such as eschar, scrub typhus is often overlooked during the initial diagnostic evaluation. We present the case of an 11-year-old child with prolonged fever and delayed diagnosis of scrub typhus to emphasize the importance of epidemiological history, early recognition, and timely treatment in endemic settings.

## Case presentation

Patient information and clinical findings

An 11-year-old boy was admitted to our hospital on December 23 with recurrent fever persisting for approximately two weeks. His symptoms included intermittent abdominal pain, abdominal distension, and episodes of vomiting. He denied diarrhea, constipation, cough, respiratory symptoms, or other focal complaints.

The patient's medical history was significant for a recent hospitalization at two first-level pediatric hospitals, where he had received five days of empirical intravenous antibiotic therapy (cefoperazone and sulbactam 1500 mg) for AUFI. Although his temperature normalized following treatment, no definitive etiology was identified, and he was discharged without a confirmed diagnosis.

Upon admission to our institution, the patient appeared fatigued but remained alert and oriented. His body temperature was 37.5°C. Physical examination revealed mild abdominal distension without tenderness, guarding, rebound tenderness, or signs of peritonitis. No skin rash, peripheral edema, lymphadenopathy, or meningeal signs were observed. Cardiovascular and respiratory examinations were unremarkable, with normal heart sounds, regular rhythm, and clear bilateral breath sounds.

Diagnostic assessment

Initial laboratory investigations showed a normal white blood cell count with a normal absolute neutrophil count, increased eosinophils, a normal platelet count, and an increased C-reactive protein level. Liver transaminases showed no clinically significant elevation. Tests for dengue virus, influenza A, influenza B, and SARS-CoV-2 were negative. Blood cultures showed no bacterial growth. Abdominal ultrasonography revealed free intraperitoneal fluid with a maximal depth of 42.5 mm. Based on the clinical presentation and imaging findings, infectious enteritis with systemic inflammatory response was initially suspected.

First Treatment

On admission on December 23, the patient was commenced on cefotaxime 1000 mg 4 ampoules/day and amikacin 500 mg × 1 time/day. From December 26, his treatment plan was added metronidazole 500 mg/day and ceftriaxone 1000 mg × 2 ampoules/day.

Clinical Progression

Despite five days of intravenous broad-spectrum antimicrobial therapy and supportive treatment, the patient continued to have persistent fever, recurrent abdominal pain, and vomiting. Repeat laboratory testing showed persistently increased inflammatory markers, a slightly decreased white blood cell count, decreased neutrophils, and persistent eosinophilia. Total immunoglobulin E was markedly increased. Follow-up abdominal ultrasonography continued to show inflammatory bowel changes with residual free intraperitoneal fluid of approximately 15 mL. Laboratory findings are summarized in Table 1.

**Table 1:** Laboratory testing G/L = 10^9^/L. "Not performed" means the test was not done. WBC: white blood cell count; NEU: neutrophils; EOS: eosinophils; PLT: platelet count; CRP: C-reactive protein; IgE: immunoglobulin E; GPT: glutamate pyruvate transaminase; GOT: glutamate oxaloacetate transaminase; AST: aspartate aminotransferase; ALT: alanine aminotransferase

Test	On admission	After 5 days of treatment	At discharge	Reference range
WBC	6.98 G/L	6.03 G/L	8.1 G/L	4.0-10.0 G/L
NEU	3.66 G/L	1.8 G/L	3.31 G/L	1.5-8.0 G/L
NEU%	52.4%	29.8%	40.8%	40-70%
EOS	0.506 G/L	0.598 G/L	0.24 G/L	0.02-0.50 G/L
EOS%	7.26%	9.91%	2.96%	0-5%
PLT	187 G/L	261 G/L	397 G/L	150-450 G/L
CRP	57.15 mg/L	55.62 mg/L	2.8 mg/L	<5 mg/L
Procalcitonin	Not performed	Not performed	0.028 ng/mL	<0.05 ng/mL
Blood culture	Negative	Not performed	Not performed	No growth/negative
IgE	Not performed	>2500 IU/mL	Not performed	200 IU/ml
GPT (ALT)	27.6 U/L	14.7 U/L	30.4 U/L	<40 U/L
GOT (AST)	44 U/L	26.8 U/L	26 U/L	<40 U/L

Given the lack of clinical improvement and persistent inflammatory findings despite antibiotic escalation, a more detailed epidemiological history was obtained on December 30. The patient's family reported that several individuals residing in the same neighborhood had recently been diagnosed with scrub typhus. Furthermore, the patient recalled sustaining an insect bite approximately one month before symptom onset while playing outdoors near his home.

Based on these epidemiological findings, serological testing for scrub typhus was performed and returned positive for *Orientia tsutsugamushi *infection, confirming the diagnosis of scrub typhus. Antimicrobial therapy was subsequently modified to include oral azithromycin 250 mg 2 times/day in addition to ongoing supportive management [6].

Outcome and Follow-Up

Following the initiation of targeted therapy, the patient's condition improved rapidly. Fever resolved, abdominal symptoms subsided, and inflammatory markers normalized. Six days after treatment modification, C-reactive protein had decreased to 2.8 mg/L and procalcitonin to 0.028 ng/mL. Repeat abdominal ultrasonography demonstrated complete resolution of the previously identified free intraperitoneal fluid. The patient was discharged on January 5 in good clinical condition, with outpatient follow-up scheduled one week later.

## Discussion

Scrub typhus, a bacterial infection caused by *Orientia tsutsugamushi*, is increasingly recognized as an important cause of fever in Asia, with an estimated one million infections occurring each year. Limited access to healthcare and the disease's nonspecific symptoms mean that many patients are undiagnosed and untreated, but the mortality from untreated scrub typhus is unknown [7]. Scrub typhus, a vector-borne rickettsiosis, is the leading treatable cause of non-malarial febrile illness in Asia. The myriad of typical and atypical features poses a clinical conundrum. In the past, the disease remained a major cause of severe, undifferentiated febrile illness in US military forces in Vietnam [8]. Scrub typhus is an important but frequently overlooked cause of AUFI in endemic regions of Asia.

The present case illustrates the diagnostic challenges of pediatric scrub typhus. The patient presented with prolonged fever, abdominal pain, vomiting, abdominal distension, and ascites, findings that initially suggested gastrointestinal infection or sepsis rather than scrub typhus. Acute undifferentiated fever is a common clinical problem in tropical countries, where several infectious diseases share overlapping manifestations. In the early stage, scrub typhus may be mistaken for dengue fever, leptospirosis, enteric fever, or bacterial sepsis because symptoms such as fever, abdominal pain, and headache are nonspecific. As a result, many patients receive empirical antimicrobial therapy before the correct diagnosis is established.

A characteristic eschar is considered an important diagnostic clue; however, its absence does not exclude the diagnosis. In a pediatric series from India involving 67 children with IgM enzyme-linked immunosorbent assay (ELISA)-confirmed scrub typhus, eschar was identified in only 46% of cases, while rash was present in 35%. Fever was universal, and gastrointestinal symptoms such as vomiting occurred in 59% of patients [9]. Our patient had prolonged fever and vomiting but lacked an identifiable eschar, rash, hepatosplenomegaly, lymphadenopathy, edema, or neurological manifestations, making the diagnosis even more challenging. Furthermore, the insect bite had resolved before admission and was only identified after repeated medical history-taking.

The epidemiological history ultimately proved critical for diagnosis. Only after clinicians re-evaluated the patient's exposure history did the family report that multiple individuals in the neighborhood had recently been diagnosed with scrub typhus and that the child had experienced an insect bite approximately one month before symptom onset. This finding highlights the importance of maintaining a high index of suspicion and obtaining a thorough epidemiological history in patients with persistent fever who fail to respond to empirical therapy. In endemic areas, overlooking exposure history may delay appropriate treatment and increase the risk of severe complications.

Although our patient experienced a favorable outcome, scrub typhus can lead to significant morbidity and mortality if left untreated. Previous studies have reported untreated mortality rates ranging from less than 1% to as high as 30%, depending on disease severity and healthcare accessibility. A systematic review from India found that scrub typhus accounted for approximately 25.3% of AUFI cases, while 17.4% of patients developed multiple organ dysfunction syndrome (MODS), 20.4% required intensive care admission, and 19.1% required mechanical ventilation. The overall case fatality rate was 6.3%, increasing to nearly 39% among patients with MODS [10]. Severe complications include acute respiratory distress syndrome (ARDS), acute kidney injury, shock, meningoencephalitis, hepatic failure, and multiorgan dysfunction.

Interestingly, our patient demonstrated persistent ascites and abdominal symptoms without evidence of severe hepatic involvement. Elevated hepatic transaminases have been associated with severe disease, delayed fever clearance, and prolonged hospitalization in children with scrub typhus [11]. However, liver enzyme levels remained within normal limits in our patient, suggesting that significant gastrointestinal manifestations may occur even in the absence of hepatic injury. This observation further emphasizes the heterogeneity of clinical presentations in pediatric scrub typhus.

The diagnosis of scrub typhus remains challenging because *Orientia tsutsugamushi *is difficult to culture, molecular testing may have limited sensitivity, and the reference standard indirect immunofluorescence assay is not widely available in many endemic settings. Consequently, clinicians often rely on a combination of clinical manifestations, epidemiological exposure, and serological testing. This diagnostic difficulty likely contributes to the under-recognition of the disease, particularly in urbanizing regions where physicians may not routinely consider scrub typhus in the differential diagnosis.

Prompt initiation of appropriate antimicrobial therapy is essential. Doxycycline and azithromycin are currently regarded as first-line agents for pediatric scrub typhus. A randomized controlled trial comparing azithromycin and doxycycline in children demonstrated comparable rates of fever defervescence, clinical recovery, and laboratory improvement, with azithromycin showing excellent tolerability [6]. A subsequent meta-analysis similarly found no significant difference in efficacy between azithromycin and other commonly used antibiotics, although the certainty of evidence remained low [12]. In our patient, the addition of azithromycin was followed by rapid clinical improvement, normalization of inflammatory markers, resolution of ascites, and complete recovery. This favorable response further supports the effectiveness of azithromycin in pediatric scrub typhus.

Recent evidence from a large randomized controlled trial in the New England Journal of Medicine involving patients aged 15 years and older with severe scrub typhus and organ involvement demonstrated that combination therapy with intravenous doxycycline and azithromycin was associated with better clinical outcomes than monotherapy with either agent alone [13]. However, evidence regarding the optimal antimicrobial regimen for pediatric patients remains limited. Most pediatric studies have focused on uncomplicated disease and have shown comparable efficacy between doxycycline and azithromycin monotherapy. Therefore, further prospective studies are needed to evaluate the role of combination therapy in children, particularly in severe cases or those with organ dysfunction, to determine whether the benefits observed in adults can be translated to the pediatric population.

## Conclusions

Early recognition of scrub typhus remains difficult because its clinical manifestations are often nonspecific and may mimic a wide range of infectious diseases. As urbanization continues to expand, physicians may be less likely to consider scrub typhus in the differential diagnosis, particularly in the absence of characteristic findings such as an eschar. This case highlights the importance of maintaining a high index of suspicion, obtaining a thorough epidemiological history, and reassessing patients who fail to respond to empirical therapy. Prompt diagnosis and targeted treatment are crucial to reducing morbidity and preventing potentially life-threatening complications.
